# Acute Liver Injury Induced by Synthetic Cannabinoid Abuse

**DOI:** 10.7759/cureus.3257

**Published:** 2018-09-04

**Authors:** Amir Shahbaz, Rafael Eduardo EEE Gaviria, Muhammad Faizan Shahid, Muhammad Awais Yasin, Attique Ashraf, Muhammad A Zaman

**Affiliations:** 1 Internal Medicine, Icahn School of Medicine at Mount Sinai/Queens Hospital Center, New York, USA; 2 Medicine, Saint Agnes Hospital, Baltimore, USA; 3 Internal Medicine, Jinnah Hospital/Allama Iqbal Medical College, Lahore, PAK; 4 Internal Medicine, Combined Military Hospital, Lahore, PAK; 5 Medicine, Allama Iqbal Medical College, Lahore, PAK; 6 Medicine, Jinnah Hospital Lahore (JHL)/Allama Iqbal Medical College (AIMC), Lahore, PAK

**Keywords:** synthetic cannabinoid, acute liver injury, n-acetylcysteine

## Abstract

Synthetic cannabinoid abuse can manifest with an array of unpredictable reactions ranging from sedation to hallucinations, psychosis, and seizures. Acute liver injury associated with the synthetic cannabinoid use is a rare complication. We present a case of a 22-year-old homeless male presented with abdominal pain and vomiting. He admitted regular synthetic cannabinoid use, and binge alcohol use once a week. Physical examination was remarkable only for mild icterus. The laboratory result shows abnormal liver functions tests. Viral, autoimmune, metabolic and other toxic etiologies of liver injury were ruled out. The acute liver injury was deemed to be secondary to synthetic cannabinoids toxicity. Spice-induced liver injury remains a diagnosis of exclusion after all other identifiable causes ruled out. Clinicians should have a high index of suspicion for synthetic cannabinoid abuse in a patient with acute hepatotoxicity who had a history of polysubstance abuse.

## Introduction

Synthetic cannabinoid (Spice/K2) abuse has been on the rise over the past decade and is responsible for an increasing number of hospital visits. Spice/K-2 abuse can manifest with an array of unpredictable reactions ranging from sedation to hallucinations, psychosis, and seizures [1]. So far two cases have been documented in the literature about acute hepatitis secondary to Spice/K2 abuse [2, 3]. We presented a case of acute liver injury secondary to Spice/K2 abuse.

## Case presentation

A 22-year-old homeless male with no known past medical history presented with a complaint of epigastric abdominal pain and non-bloody bilious vomiting for last two days. He was taking amphetamine and cocaine but quit nine months ago. He admitted recent regular Spice/K2 use. He starts taking Spice/K-2 one week prior to the onset of symptoms. Physical examination was unremarkable except mild ictrius. Abdominal examination was normal. Aspartate aminotransferase (AST) 712 IU/L, and alanine aminotransferase (ALT) 1764 IU/L were elevated. Total bilirubin and direct bilirubin were 3.8 mg/dl and 1 mg/dl, respectively. Hepatitis B surface antigen (HBsAg), anti-hepatitis C virus (HCV), antinuclear antibodies (ANA), anti-smooth muscle antibodies (ASMA), and anti-liver-kidney microsome-1 antibodies (ALKM-1) were negative. Blood alcohol level was undetectable, and urine toxicology was positive for cannabinoids and barbiturates. Blood work ruled out viral, autoimmune, metabolic and other toxic etiologies of liver injury. Therefore, patient acute liver injury was deemed to be secondary to Spice/K2 toxicity. N-acetyl cysteine initiated for imminent acute liver failure with clinical and biochemical improvement observed over a course of one week. The trend of liver enzymes during hospitalization is shown in Table 1.

**Table 1:** Hepatic transaminases over the course of hospitalization. AST: Aspartate aminotransferase; ALT: Alanine aminotransferase.

	Day 1	Day 2	Day 3	Day 4	Day 5	Day 6	Day 7	Day 8
AST	712 IU/L	1335 IU/L	940 IU/L	588 IU/L	230 IU/L	131 IU/L	79 IU/L	38 IU/L
ALT	1764 IU/L	2436 IU/L	1327 IU/L	719 IU/L	288 IU/L	161 IU/L	108 IU/L	48 IU/L
Total Bilirubin	3.8 mg/dl	3.7 mg/dl	2.7 mg/dl	2.3 mg/dl	1.6 mg/dl	1.2 mg/dl	0.8 mg/dl	0.8 mg/dl

The patient discharged in stable clinical condition and on follow-up visit after four weeks he reported no further symptoms and liver function tests were within normal range. The patient counseled about abstinence from drugs of abuse, and he agreed to consider joining the outpatient drug rehabilitation program.

## Discussion

Tetrahydrocannabinol (THC) is the active compound found in Cannabis sativa and is responsible for marijuana’s psychoactive effects. By partially binding to G-protein-coupled cannabinoid receptors type 1 and 2 (CB1 and CB2) it causes psychological and physiologic changes that include alterations of perception and mood, and hemodynamic changes such as tachycardia and hypotension [4]. Synthetic cannabinoid initially manufactured for understanding the body’s endocannabinoid system and unmasking its potential therapeutic effects. However, by the year 2000, they began to sell over the internet as natural, cheap, psychoactive cannabis under names such as SPICE and K2 [2]. They are cheaper than regular marijuana and are not detectable by routine toxicology screening [5]. The synthetic cannabinoid is an oversimplification for this group of psychoactive designer drugs. They are synthesized by spraying dried natural herbs with one or more compounds from several classes of drugs such as benzoylindoles, naphthoylindoles, phenylacetylindoles, and cyclohexylphenols [2]. Adding to the potentially deleterious effects is the variability in the amount and mixture of active compound that can be present per product [6]. Unlike THC present in the Cannabis sativa plant, the compounds in Spice/K2 act as high-potency full agonists at the brain CB1 receptors and produce an exaggerated change in perception and mood, and a more robust change in hemodynamics [7]. The constituents of Spice/K2 also change with time, mainly because the producers want to be one step ahead of legislation [8]. Spice/K2 causes an alteration in mood, perception, thinking, memory, and attention, as well as changes in the neurological, cardiovascular, and gastrointestinal function similar to those caused by THC, but they vary in both spectrum and intensity [4]. In the review of the literature, we found only two case reports of acute liver injury secondary to Spice/K2 abuse [2,3].

In our patient, there were no other identifiable agents that could explain his hepatic injury except Spice/K2 intake. Urine toxicology positive for cannabinoids, and barbiturates along with our patient’s confession of Spice/K2 use accepted as verification. Unfortunately, our hospital did not have any assay that would specifically confirm the ingestion of Spice/K2. The exact mechanism of acute liver injury is unknown. Dose-related cumulative oxidative hepatocellular necrosis theorized as a possible mechanism. Symptoms occur immediately, minutes, or hours after use and may be transient or last for hours. The association of symptoms with Spice/K2 use, therefore, is easily missed [2]. Empiric use of N-acetylcysteine should be considered because it is relatively safe, and leads to a favorable clinical outcome. Spice/K2-induced liver injury remains a diagnosis of exclusion after all other identifiable causes ruled out.

## Conclusions

Spice/K2 recently emerged as a threat to public health and safety. Patient with acute hepatotoxicity that has a history of polysubstance abuse should be evaluated for synthetic cannabinoid use. More studies are needed to evaluate the utility of N-acetylcysteine in the setting of Spice/K2-induced liver injury.
